# Supplementary material for: Perceptions of green space usage, abundance, and quality of green space were associated with better mental health during the COVID-19 pandemic among residents of Denver
Source: PLoS One. 2022 Mar 2;17(3):e0263779. doi: 10.1371/journal.pone.0263779 (PMC8890647; doi:10.1371/journal.pone.0263779)
Supplement: S1 File — (PDF) [file pone.0263779.s001.pdf]

Questionnaire on Neighborhood Green Space Perception

Below we include the questions from our survey that were used in the analysis presented in the paper tiled “Perceptions of green space usage, abundance, and quality of green space were associated with better mental health during the COVID-19 pandemic among residents of Denver” by Reid, Rieves, and Carlton.

Contents

Introduction 1

NEIGHBORHOOD INFORMATION 2

SOCIAL CAPITAL & SOCIAL COHESION 3

COHEN PERCEIVED STRESS SCALE (4-item version) 4

CES-D DEPRESSION 5

MMPI Anxiety Scale 6

GENERAL & MENTAL HEALTH 8

GREEN SPACE EXPOSURE 10

ENVIRONMENTAL VIEWS 12

Neighborhood Violence 14

DEMOGRAPHIC Information 15

COVID-19 Questions 17

Introduction

This survey is being conducted by researchers at the University of Colorado Boulder. We're conducting an important study to understand how neighborhoods affect health within Denver neighborhoods. You have been selected as a representative of your neighborhood.

All answers you give will be confidential. You don’t have to give me any personal identifying information such as your full name.

We will first ask some questions to determine your eligibility for the study. If you are eligible, you will then be provided with information on the survey and asked for your consent to participate. Should you choose to participate, you will then be able to continue on to the survey.

NEIGHBORHOOD INFORMATION

In this section, you will be asked information about where you live.

1. Please draw the outline of the area you consider your neighborhood.

[This will take the survey respondent to Google Maps and they will be provided with information on how to draw their neighborhood and save that polygon for the survey]

2. How did you decide where to draw the boundaries of your neighborhood?

3. How long have you lived in your neighborhood?

- ☐ 1 Less than 1 Year
- ☐ 2 1-5 years
- ☐ 3 5-10 years
- ☐ 4 More than 10 years

4. What is your address? (This information will be used to better understand your neighborhood location. If you are uncomfortable sharing your address, please provide the nearest cross-streets (intersection) to your home)

____

5. What is your residential zip code? _______

6. Where did you grow up?

COHEN PERCEIVED STRESS SCALE (4-item version)

We asked participants to answer each of the questions from the four-item Cohen Perceived Stress Scale.

- Cohen S, Kamarck T, Mermelstein R. 1983. A global measure of perceived stress. J Health Soc Behav 24:385-396. Scale available: http://www.psy.cmu.edu/~scohen/PSS4.html

CES-D DEPRESSION

We asked participants to answer each of the questions from the 10-item Center for Epidemiological Studies Depression Scale.

- Radloff LS. 1977. The CES-D scale: A self -report depression scale for research in the general population. Applied Psychological Measurement 1:385-401.
- Irwin M, Artin KH, Oxman MN. 1999. Screening depression in the older adult: criterion validity of the 10-item Center for Epidemiological Studies Depression Scale (CES-D). Arch Intern Med 159:1701-1704.

MMPI Anxiety Scale

We asked participants to answer each of the questions from the Minnesota Multiphasic Personality Inventory-2 Scale.

- Butcher JN, Dahlstrom WG, Graham JR, Tellegen A, & Kaemmer B. *MMPI-2: Minnesota Multiphasic Personality Inventory-2. Manual for administration and scoring*. Minneapolis, MN: University ofMinnesota Press: 1989.

GENERAL & MENTAL HEALTH

We asked participants to answer nine questions reproduced from the NYC Department of Health and Mental Hygiene's 2009 Community Health Survey.

- Department of Health and Mental Hygiene (NYC DOHMH). 2009 Community Health Survey Questionnaire. Available: https://www1.nyc.gov/site/doh/data/data-sets/community-health-survey-public-use-data.page

We also asked participants to answer a tenth question, about their weekly level of moderate physical activity, which was reproduced from a survey administered by de Jong et al. (2012).

- de Jong K, Albin M, Skarback E, Grahn P, Bjork J. 2012. Perceived green qualities were associated with neighborhood satisfaction, physical activity, and general health: results from a cross- sectional study in suburban and rural Scania, southern Sweden. Health Place 18:1374–80; doi: 10.1016/j.healthplace.2012.07.001.

GREEN SPACE EXPOSURE

For the following questions, the term "green space" means any area with natural vegetation. This can include parks, yards, grassy areas, street trees, green roofs, cemeteries, etc.[1]

Q1. For each of the following statements about your home neighborhood, state whether you strongly agree, agree, disagree, or strongly disagree.[2]

1. There is a lot of vegetation/greenery in my neighborhood
2. I can see vegetation/greenery from my home
3. The nearest vegetated park/green space is easy for me to access
4. I spend a lot of time in spaces with natural vegetation
5. The green spaces near my home are very high in quality

RESPONSE OPTIONS:

1. Strongly Disagree
2. Disagree
3. Agree
4. Strongly Agree

---

[1] We adapted the definition of "green space" used for this question from Sugiyama et al. (2010).
Leslie, E., Sugiyama, T., Ierodiaconou, D., & Kremer, P. (2010). Perceived and objectively measured greenness of neighbourhoods: Are they measuring the same thing? *Landscape and Urban Planning*, *95*(1), 28–33. https://doi.org/10.1016/j.landurbplan.2009.11.002

[2] We asked participants to answer five questions about their perceptions of greenspace within their neighborhoods, which were adapted from Dzhambov et al. (2018).
Dzhambov AM, Markevych I, Hartig T, Tilov B, Arabadzhiev Z, Stoyanov D, Gatseva P, Dimitrova DD. Multiple pathways link urban green- and bluespace to mental health in young adults. Environ Res. 2018 Oct;166:223-233. doi: 10.1016/j.envres.2018.06.004.

DEMOGRAPHIC Information

1. Age: _____
2. Sex:
   a. Male
   b. Female
3. Ethnicity
   a. Hispanic/Latino
   b. NOT Hispanic/Latino
4. Race
   a. White/Caucasian
   b. Black/African American
   c. Asian/Pacific Islander
   d. Native American
   e. Other: _____
5. Marital Status:
   a. Married/living as married
   b. Separated
   c. Divorced
   d. Widowed
   e. Single, never married
6. Which one of the following BEST describes your employment status?
   a. Employed full-time or self-employed
   b. Employed part-time
   c. Out of work and looking for wrok
   d. Ouf of work but not currently looking for work
   e. A homemaker
   f. A student
   g. Retired
   h. Unable to work
7. Occupation: _____________
8. What kind of health insurance do you have? (CHECK ALL THAT APPLY)
   a. Private, through my employer or purachased through an exchance
   b. Medicare
   c. Medicaid
   d. Self-pay (I pay for all health care out of my own funds)
   e. Other: _______
   f. No insurance
9. Education: Highest level of school you have completed?
   a. Less than high school/ Did not complete high school
   b. High school or equivalent
   c. Some college

d. Associate’s / 2-year degree
e. Bachelor’s / 4-year degree
f. Graduate or professional degree (Ph.D., Masters, J.D., M.D., etc.)

10. What is your estimated annual household income?
    a. Less than $25,000
    b. $25,000 - $50,000
    c. $50,000 - $75,000
    d. $75,000 - $100,000
    e. $100,000 - $150,000
    f. Greater than $150,000

COVID-19 Questions

*This section will be coded into the Qualtrics survey such that once a participant gets to this point in their survey, they cannot change their responses to the previous questions.

We asked participants to answer nine questions reproduced from *Social Psychological Survey of COVID-19: Coronavirus Perceived Threat, Government Response, Impacts, and Experiences Questionnaires*.

- Conway, L. G., III, Woodard, S. R., & Zubrod, A. (2020, April 7). Social Psychological Measurements of COVID-19: Coronavirus Perceived Threat, Government Response, Impacts, and ExperiencesQuestionnaires. https://doi.org/10.31234/osf.io/z2x9a
- The paper can be accessed directly here: https://psyarxiv.com/z2x9a/

We also asked participants to answer one question from *The Epidemic – Pandemic Impacts Inventory (EPII)*.

- Grasso, D.J., Briggs-Gowan, M.J., Ford, J.D., & Carter, A.S. (2020). The Epidemic – Pandemic Impacts Inventory (EPII).
- The EPII can be directly accessed here: https://www.google.com/url?sa=t&rct=j&q=&esrc=s&source=web&cd=&ved=2ahUKEwib4b7L_N71AhViLH0KHdxFBl8QFnoECAwQAQ&url=https%3A%2F%2Fwww.phenxtoolkit.org%2Ftoolkit_content%2FPDF%2FGrasso_EPII.pdf&usg=AOvVaw19oDacuvGB7mgxtcqiaSJi

We also asked participants to answer one question that we created specifically for this survey. We matched the scoring to the other COVID-19 questions that we asked, in which scales were presented with options from 1-7 anchored by “1 = not true of me at all” and “7 = very true of me.”

Q10. I have spent more time outside in parks, on trails, and near nature in the past month compared to the same time last year.
