# Supplementary material for: Perceptions of green space usage, abundance, and quality of green space were associated with better mental health during the COVID-19 pandemic among residents of Denver
Source: PLoS One. 2022 Mar 2;17(3):e0263779. doi: 10.1371/journal.pone.0263779 (PMC8890647; doi:10.1371/journal.pone.0263779)
Supplement: S1 Table — (DOCX) [file pone.0263779.s002.docx]

|  | **PSS Stress**,  N = 807 | | | **CES-D-10 Depression**,  N = 801 | | | **MMPI-2 Anxiety**,  N = 785 | | |
| --- | --- | --- | --- | --- | --- | --- | --- | --- | --- |
| **Sociodemographic measure** | **Beta** | **95% CI** | **p-value** | **Beta** | **95% CI** | **p-value** | **Beta** | **95% CI** | **p-value** |
| Sex | | | | | | | | | |
| Female | — | — |  | — | — |  | — | — |  |
| Male | -0.53 | -0.93, -0.12 | **0.011** | -0.69 | -1.42, 0.04 | 0.064 | -1.25 | -2.84, 0.34 | 0.124 |
| Income | | | | | | | | | |
| Less than $25,000 | — | — |  | — | — |  | — | — |  |
| $25,000 to $50,000 | -0.32 | -1.07, 0.44 | 0.415 | -1.18 | -2.55, 0.19 | 0.091 | -1.11 | -4.11, 1.90 | 0.472 |
| $50,000 to $75,000 | -0.63 | -1.40, 0.14 | 0.111 | -2.37 | -3.76, -0.97 | **<0.001** | -4.64 | -7.68, -1.60 | **0.003** |
| $75,000 to $100,000 | -0.81 | -1.59, -0.03 | **0.042** | -2.76 | -4.16, -1.36 | **<0.001** | -4.25 | -7.31, -1.19 | **0.007** |
| $100,000 to $150,000 | -1.44 | -2.17, -0.71 | **<0.001** | -2.89 | -4.21, -1.58 | **<0.001** | -5.77 | -8.66, -2.89 | **<0.001** |
| Greater than $150,000 | -1.88 | -2.60, -1.15 | **<0.001** | -3.87 | -5.17, -2.56 | **<0.001** | -7.97 | -10.84, -5.11 | **<0.001** |
| Race | | | | | | | | | |
| White | — | — |  | — | — |  | — | — |  |
| Asian/Pacific Islander | 0.53 | -0.99, 2.05 | 0.493 | 1.21 | -1.52, 3.93 | 0.385 | 2.05 | -3.87, 7.98 | 0.497 |
| Black/African American | 0.67 | -0.34, 1.69 | 0.194 | 0.27 | -1.58, 2.13 | 0.773 | 1.59 | -2.51, 5.69 | 0.447 |
| Multiracial | 0.90 | -0.21, 2.00 | 0.112 | 1.39 | -0.59, 3.37 | 0.168 | 2.53 | -1.95, 7.00 | 0.269 |
| Native American | 0.38 | -1.00, 1.76 | 0.590 | 0.33 | -2.22, 2.89 | 0.798 | 2.44 | -3.12, 7.99 | 0.390 |
| Ethnicity | | | | | | | | | |
| Not Hispanic/Latino | — | — |  | — | — |  | — | — |  |
| Hispanic/Latino | 0.26 | -0.31, 0.84 | 0.370 | 0.93 | -0.11, 1.97 | 0.080 | 3.33 | 1.06, 5.59 | **0.004** |
| Age |  |  |  |  |  |  |  |  |  |
| 18 to 29 | — | — |  | — | — |  | — | — |  |
| 30 to 49 | -0.34 | -0.85, 0.16 | 0.184 | -1.11 | -2.03, -0.18 | **0.019** | -2.09 | -4.03, -0.14 | **0.036** |
| 50 to 64 | -1.20 | -1.81, -0.58 | **<0.001** | -2.06 | -3.19, -0.94 | **<0.001** | -6.56 | -8.93, -4.19 | **<0.001** |
| 65+ | -2.43 | -3.05, -1.81 | **<0.001** | -4.07 | -5.21, -2.94 | **<0.001** | -11.18 | -13.58, -8.79 | **<0.001** |
| Cohabitation status | | | | | | | | | |
| Partnered | — | — |  | — | — |  | — | — |  |
| Unpartnered | 1.02 | 0.62, 1.41 | **<0.001** | 1.98 | 1.27, 2.69 | **<0.001** | 3.66 | 2.10, 5.21 | **<0.001** |
| Educational attainment | | | | | | | | | |
| BA or higher | — | — |  | — | — |  | — | — |  |
| Less than BA | 1.13 | 0.69, 1.57 | **<0.001** | 1.79 | 0.99, 2.58 | **<0.001** | 4.85 | 3.13, 6.57 | **<0.001** |
| Looking for work? | | | | | | | | | |
| Not looking | — | — |  | — | — |  | — | — |  |
| Looking - part-time | 1.04 | 0.27, 1.80 | **0.008** | 1.54 | 0.17, 2.92 | 0.028 | 3.08 | 0.06, 6.11 | 0.046 |
| Looking | 2.38 | 1.57, 3.19 | **<0.001** | 3.92 | 2.46, 5.37 | **<0.001** | 9.32 | 6.16, 12.48 | **<0.001** |
| Insurance status | | | | | | | | | |
| Insured | — | — |  | — | — |  | — | — |  |
| Not insured | 1.45 | 0.83, 2.07 | **<0.001** | 2.48 | 1.36, 3.61 | **<0.001** | 6.72 | 4.32, 9.12 | **<0.001** |
