# Supplementary material for: Perceptions of green space usage, abundance, and quality of green space were associated with better mental health during the COVID-19 pandemic among residents of Denver
Source: PLoS One. 2022 Mar 2;17(3):e0263779. doi: 10.1371/journal.pone.0263779 (PMC8890647; doi:10.1371/journal.pone.0263779)
Supplement: S2 Table — (DOCX) [file pone.0263779.s003.docx]

| Category | Study observation | Denver population | Study-Denver p-value (Chi-squared test) | Neighborhood population | Study-Neighborhood p-value (Chi-squared test) |
| --- | --- | --- | --- | --- | --- |
| **Sex** | | | | | |
| Female | 526 (57.7%) | 49.9% | **<0.001** | 49.3% | **<0.001** |
| Male | 385 (42.3%) | 50.1% |  | 50.7% |  |
| **Age group** | | | | | |
| 20 to 34 years | 320 (35.7%) | 37.3% | **0.016** | 31.3% | **0.004** |
| 35 to 54 years | 296 (33.0%) | 35.1% |  | 25.8% |  |
| 55 to 64 years | 114 (12.7%) | 12.8% |  | 10.6% |  |
| 65+ | 166 (18.5%) | 14.8% |  | 11.5% |  |
| **Ethnicity** | | | | | |
| Not Hispanic/Latino | 779 (85.5%) | 70.1% | **<0.001** | 63.7% | **<0.001** |
| Hispanic/Latino | 132 (14.5%) | 29.9% |  | 36.3% |  |
| **Educational attainment** | | | | | |
| BA or higher | 666 (73.2%) | 49.4% | **<0.001** | 41.7% | **<0.001** |
| Less than BA | 244 (26.8%) | 50.6% |  | 58.3% |  |
| **Income** | | | | | |
| Greater than $150,000 | 191 (21.4%) | 18.1% | **<0.001** | 15.5% | **<0.001** |
| $100,000 to $150,000 | 181 (20.3%) | 15.7% |  | 14.3% |  |
| $25,000 to $50,000 | 148 (16.6%) | 12.5% |  | 12.1% |  |
| $50,000 to $75,000 | 140 (15.7%) | 17.3% |  | 17.0% |  |
| $75,000 to $100,000 | 135 (15.1%) | 19.2% |  | 20.3% |  |
| Less than $25,000 | 98 (11.0%) | 17.2% |  | 20.9% |  |
| **Race** | | | | | |
| White | 780 (85.6%) | 76.1% | **<0.001** | 74.0% | **<0.001** |
| Black/African American | 33 (3.6%) | 9.2% |  | 11.2% |  |
| Multiracial | 29 (3.2%) | 3.8% |  | 3.0% |  |
| Asian/Pacific Islander | 16 (1.8%) | 4.0% |  | 3.3% |  |
| Native American | 18 (2.0%) | 0.9% |  | 0.9% |  |
| NA | 35 (3.8%) | 6.1% |  | 7.7% |  |
| **Insurance status** | | | | | |
| Insured | 806 (88.5%) | 90.7% | **0.021** | 88.3% | 0.870 |
| Not insured | 105 (11.5%) | 9.3% |  | 11.7% |  |
