# Supplementary material for: Perceptions of green space usage, abundance, and quality of green space were associated with better mental health during the COVID-19 pandemic among residents of Denver
Source: PLoS One. 2022 Mar 2;17(3):e0263779. doi: 10.1371/journal.pone.0263779 (PMC8890647; doi:10.1371/journal.pone.0263779)
Supplement: S3 Table — (DOCX) [file pone.0263779.s004.docx]

|  |  | **COVID-19 time period** | |
| --- | --- | --- | --- |
| **COVID-19 impact variables***^1^* | **Total**  N=431*^2^* | **Reopening period**  N=177 (41%)*^2^* | **Second wave**  N=254 (59%)*^2^* |
| **COVID-19 has impacted me negatively from a financial point of view** | | | |
| 1 = Not true of me at all | 147 (34%) | 69 (39%) | 78 (31%) |
| 2 | 68 (16%) | 28 (16%) | 40 (16%) |
| 3 | 33 (7.7%) | 15 (8.5%) | 18 (7.1%) |
| 4 | 31 (7.2%) | 10 (5.6%) | 21 (8.3%) |
| 5 | 57 (13%) | 23 (13%) | 34 (13%) |
| 6 | 28 (6.5%) | 10 (5.6%) | 18 (7.1%) |
| 7 = Very true of me | 67 (16%) | 22 (12%) | 45 (18%) |
| **I have lost job-related income due to COVID-19** | | | |
| 1 = Not true of me at all | 232 (54%) | 106 (60%) | 126 (50%) |
| 2 | 36 (8.4%) | 15 (8.5%) | 21 (8.3%) |
| 3 | 17 (3.9%) | 5 (2.8%) | 12 (4.7%) |
| 4 | 12 (2.8%) | 1 (0.6%) | 11 (4.3%) |
| 5 | 19 (4.4%) | 9 (5.1%) | 10 (3.9%) |
| 6 | 25 (5.8%) | 5 (2.8%) | 20 (7.9%) |
| 7 = Very true of me | 90 (21%) | 36 (20%) | 54 (21%) |
| **I have had a hard time getting needed resources (food, toilet paper) due to COVID-19** | | | |
| 1 = Not true of me at all | 194 (45%) | 76 (43%) | 118 (46%) |
| 2 | 94 (22%) | 39 (22%) | 55 (22%) |
| 3 | 53 (12%) | 20 (11%) | 33 (13%) |
| 4 | 36 (8.4%) | 19 (11%) | 17 (6.7%) |
| 5 | 29 (6.7%) | 10 (5.6%) | 19 (7.5%) |
| 6 | 11 (2.6%) | 6 (3.4%) | 5 (2.0%) |
| 7 = Very true of me | 14 (3.2%) | 7 (4.0%) | 7 (2.8%) |
| **I had to continue to work even though I was in close contact with people who might be infected (e.g. customers, patients, co-workers)** | | | |
| 1 = Not true of me at all | 263 (61%) | 120 (68%) | 143 (56%) |
| 2 | 41 (9.5%) | 18 (10%) | 23 (9.1%) |
| 3 | 17 (3.9%) | 5 (2.8%) | 12 (4.7%) |
| 4 | 14 (3.2%) | 5 (2.8%) | 9 (3.5%) |
| 5 | 14 (3.2%) | 6 (3.4%) | 8 (3.1%) |
| 6 | 17 (3.9%) | 2 (1.1%) | 15 (5.9%) |
| 7 = Very true of me | 65 (15%) | 21 (12%) | 44 (17%) |
| **I have been diagnosed with COVID-19** | | | |
| *Negative* | 420 (97%) | 174 (98%) | 246 (97%) |
| *Positive* | 11 (2.6%) | 3 (1.7%) | 8 (3.1%) |
| **I have had COVID-19-like symptoms at some point in the last two months** | | | |
| 1 = Not true of me at all | 333 (77%) | 139 (79%) | 194 (76%) |
| 2 | 30 (7.0%) | 10 (5.6%) | 20 (7.9%) |
| 3 | 25 (5.8%) | 8 (4.5%) | 17 (6.7%) |
| 4 | 8 (1.9%) | 3 (1.7%) | 5 (2.0%) |
| 5 | 15 (3.5%) | 10 (5.6%) | 5 (2.0%) |
| 6 | 2 (0.5%) | 0 (0%) | 2 (0.8%) |
| 7 = Very true of me | 18 (4.2%) | 7 (4.0%) | 11 (4.3%) |
| **I have been in close proximity with someone who has been diagnosed with COVID-19** | | | |
| 1 = Not true of me at all | 256 (59%) | 116 (66%) | 140 (55%) |
| 2 | 42 (9.7%) | 15 (8.5%) | 27 (11%) |
| 3 | 28 (6.5%) | 9 (5.1%) | 19 (7.5%) |
| 4 | 26 (6.0%) | 6 (3.4%) | 20 (7.9%) |
| 5 | 13 (3.0%) | 5 (2.8%) | 8 (3.1%) |
| 6 | 9 (2.1%) | 4 (2.3%) | 5 (2.0%) |
| 7 = Very true of me | 57 (13%) | 22 (12%) | 35 (14%) |
| **I have been in close proximity with someone who has had COVID-19-like symptoms in the last two months** | | | |
| 1 = Not true of me at all | 292 (68%) | 132 (75%) | 160 (63%) |
| 2 | 35 (8.1%) | 13 (7.3%) | 22 (8.7%) |
| 3 | 12 (2.8%) | 4 (2.3%) | 8 (3.1%) |
| 4 | 17 (3.9%) | 2 (1.1%) | 15 (5.9%) |
| 5 | 7 (1.6%) | 3 (1.7%) | 4 (1.6%) |
| 6 | 8 (1.9%) | 1 (0.6%) | 7 (2.8%) |
| 7 = Very true of me | 60 (14%) | 22 (12%) | 38 (15%) |
| **I spend a huge percentage of my time trying to find updates online or on TV about COVID-19** | | | |
| 1 = Not true of me at all | 149 (35%) | 68 (38%) | 81 (32%) |
| 2 | 87 (20%) | 37 (21%) | 50 (20%) |
| 3 | 83 (19%) | 32 (18%) | 51 (20%) |
| 4 | 58 (13%) | 22 (12%) | 36 (14%) |
| 5 | 34 (7.9%) | 13 (7.3%) | 21 (8.3%) |
| 6 | 9 (2.1%) | 1 (0.6%) | 8 (3.1%) |
| 7 = Very true of me | 11 (2.6%) | 4 (2.3%) | 7 (2.8%) |
| **I have spent more time outside on parks, on trails, and near nature in the past month compared to the same time last year** | | | |
| 1 = Not true of me at all | 126 (29%) | 55 (31%) | 71 (28%) |
| 2 | 46 (11%) | 19 (11%) | 27 (11%) |
| 3 | 56 (13%) | 22 (12%) | 34 (13%) |
| 4 | 59 (14%) | 21 (12%) | 38 (15%) |
| 5 | 52 (12%) | 22 (12%) | 30 (12%) |
| 6 | 34 (7.9%) | 10 (5.6%) | 24 (9.4%) |
| 7 = Very true of me | 58 (13%) | 28 (16%) | 30 (12%) |

*^1^* Where 1 = Not true of me at all and 7 = Very true of me

*^2^* Reopening (5/9/20-10/12/20); Second wave (10/13/20-1/2/21). No respondents completed COVID-19 questions in Before COVID-19 (before 3/12/21) or Stay at home (3/13/20-5/8/20) periods.
