# Supplementary material for: Perceptions of green space usage, abundance, and quality of green space were associated with better mental health during the COVID-19 pandemic among residents of Denver
Source: PLoS One. 2022 Mar 2;17(3):e0263779. doi: 10.1371/journal.pone.0263779 (PMC8890647; doi:10.1371/journal.pone.0263779)
Supplement: S4 Table — (DOCX) [file pone.0263779.s005.docx]

|  | **PSS Stress**,  N = 807 | | | **CES-D-10 Depression**,  N = 801 | | | **MMPI-2 Anxiety**,  N = 785 | | |
| --- | --- | --- | --- | --- | --- | --- | --- | --- | --- |
| **Greenspace measure** | **Beta** | **95% CI** | **p-value** | **Beta** | **95% CI** | **p-value** | **Beta** | **95% CI** | **p-value** |
| “There is a lot of vegetation/greenery in my neighborhood” | | | | | | | | | |
| *Strongly Disagree* | — | — |  | — | — |  | — | — |  |
| *Disagree* | -0.50 | -1.29, 0.30 | 0.224 | -1.43 | -2.84, -0.01 | **0.049** | -1.67 | -4.70, 1.37 | 0.282 |
| *Agree* | -0.72 | -1.44, 0.01 | 0.052 | -2.42 | -3.71, -1.14 | **<0.001** | -3.76 | -6.50, -1.02 | **0.007** |
| *Strongly Agree* | -1.65 | -2.41, -0.90 | **<0.001** | -4.02 | -5.36, -2.67 | **<0.001** | -9.04 | -11.91, -6.17 | **<0.001** |
| “I can see vegetation/greenery from my home” | | | | | | | | | |
| *Strongly Disagree* | — | — |  | — | — |  | — | — |  |
| *Disagree* | -0.45 | -1.32, 0.42 | 0.310 | -0.18 | -1.72, 1.37 | 0.822 | -0.87 | -4.21, 2.46 | 0.608 |
| *Agree* | -0.89 | -1.66, -0.12 | **0.023** | -1.89 | -3.25, -0.53 | **0.007** | -3.47 | -6.42, -0.53 | **0.021** |
| *Strongly Agree* | -1.81 | -2.59, -1.03 | **<0.001** | -3.42 | -4.80, -2.04 | **<0.001** | -8.75 | -11.75, -5.75 | **<0.001** |
| “The nearest vegetated park/green space is easy for me to access” | | | | | | | | | |
| *Strongly Disagree* | — | — |  | — | — |  | — | — |  |
| *Disagree* | -0.06 | -1.42, 1.31 | 0.934 | -2.39 | -4.85, 0.08 | 0.059 | -4.36 | -9.58, 0.86 | 0.102 |
| *Agree* | -0.61 | -1.75, 0.54 | 0.300 | -3.22 | -5.28, -1.16 | **0.002** | -5.64 | -10.03, -1.25 | **0.012** |
| *Strongly Agree* | -1.43 | -2.57, -0.29 | **0.014** | -4.28 | -6.32, -2.23 | **<0.001** | -9.73 | -14.09, -5.38 | **<0.001** |
| “I spend a lot of time in spaces with natural vegetation” | | | | | | | | | |
| *Strongly Disagree* | — | — |  | — | — |  | — | — |  |
| *Disagree* | -0.74 | -1.62, 0.13 | 0.096 | -3.13 | -4.69, -1.58 | **<0.001** | -7.12 | -10.43, -3.80 | **<0.001** |
| *Agree* | -1.17 | -2.01, -0.33 | **0.006** | -4.39 | -5.89, -2.90 | **<0.001** | -10.20 | -13.38, -7.02 | **<0.001** |
| *Strongly Agree* | -1.76 | -2.63, -0.89 | **<0.001** | -5.25 | -6.80, -3.70 | **<0.001** | -12.85 | -16.17, -9.54 | **<0.001** |
| “The green spaces near my home are very high quality” | | | | | | | | | |
| *Strongly Disagree* | — | — |  | — | — |  | — | — |  |
| *Disagree* | -0.33 | -1.05, 0.40 | 0.377 | -1.07 | -2.37, 0.22 | 0.103 | -2.82 | -5.58, -0.07 | 0.045 |
| *Agree* | -0.49 | -1.17, 0.20 | 0.164 | -2.10 | -3.32, -0.88 | **<0.001** | -5.53 | -8.15, -2.92 | **<0.001** |
| *Strongly Agree* | -1.60 | -2.33, -0.87 | **<0.001** | -3.95 | -5.25, -2.64 | **<0.001** | -10.52 | -13.32, -7.72 | **<0.001** |
| NAIP NDVI – 300 m buffer | -4.79 | -7.36, -2.22 | **<0.001** | -13.40 | -17.97, -8.82 | **<0.001** | -33.20 | -43.14, -23.27 | **<0.001** |
| NAIP NDVI – 500 m buffer | -4.63 | -7.34, -1.92 | **<0.001** | -14.62 | -19.43, -9.80 | **<0.001** | -34.75 | -45.23, -24.28 | **<0.001** |
