# Supplementary material for: Perceptions of green space usage, abundance, and quality of green space were associated with better mental health during the COVID-19 pandemic among residents of Denver
Source: PLoS One. 2022 Mar 2;17(3):e0263779. doi: 10.1371/journal.pone.0263779 (PMC8890647; doi:10.1371/journal.pone.0263779)
Supplement: S5 Table — (DOCX) [file pone.0263779.s006.docx]

|  | **PSS and ethnicity** | | | **PSS, sex, and ethnicity** | | |
| --- | --- | --- | --- | --- | --- | --- |
| **Greenspace measure** | **Beta** | **95% CI** | **p-value** | **Beta** | **95% CI** | **p-value** |
| “There is a lot of vegetation/greenery in my neighborhood” | | | | | | |
| *Strongly Disagree* | — | — |  | — | — |  |
| *Disagree* | -0.52 | -1.28, 0.23 | 0.173 | -0.52 | -1.27, 0.23 | 0.174 |
| *Agree* | -0.45 | -1.13, 0.23 | 0.200 | -0.42 | -1.10, 0.26 | 0.224 |
| *Strongly Agree* | -0.66 | -1.39, 0.07 | 0.078 | -0.65 | -1.38, 0.08 | 0.081 |
| “I can see vegetation/greenery from my home” | | | | | | |
| *Strongly Disagree* | — | — |  | — | — |  |
| *Disagree* | -0.60 | -1.42, 0.22 | 0.153 | -0.61 | -1.44, 0.21 | 0.143 |
| *Agree* | -0.65 | -1.38, 0.08 | 0.080 | -0.66 | -1.38, 0.07 | 0.076 |
| *Strongly Agree* | -0.76 | -1.52, 0.00 | 0.051 | -0.77 | -1.53, -0.01 | **0.048** |
| “The nearest vegetated park/green space is easy for me to access” | | | | | | |
| *Strongly Disagree* | — | — |  | — | — |  |
| *Disagree* | 0.00 | -1.30, 1.31 | 0.994 | 0.00 | -1.30, 1.30 | 0.997 |
| *Agree* | -0.42 | -1.52, 0.67 | 0.451 | -0.40 | -1.50, 0.69 | 0.468 |
| *Strongly Agree* | -0.77 | -1.87, 0.32 | 0.167 | -0.75 | -1.84, 0.34 | 0.180 |
| “I spend a lot of time in spaces with natural vegetation” | | | | | | |
| *Strongly Disagree* | — | — |  | — | — |  |
| *Disagree* | -0.46 | -1.28, 0.37 | 0.275 | -0.54 | -1.37, 0.28 | 0.197 |
| *Agree* | -0.68 | -1.48, 0.12 | 0.095 | -0.77 | -1.56, 0.03 | 0.060 |
| *Strongly Agree* | -0.87 | -1.71, -0.04 | **0.041** | -0.99 | -1.83, -0.15 | **0.021** |
| “The green spaces near my home are very high quality” | | | | | | |
| *Strongly Disagree* | — | — |  | — | — |  |
| *Disagree* | -0.18 | -0.86, 0.49 | 0.596 | -0.20 | -0.88, 0.47 | 0.559 |
| *Agree* | -0.05 | -0.70, 0.60 | 0.883 | -0.01 | -0.66, 0.63 | 0.971 |
| *Strongly Agree* | -0.47 | -1.19, 0.25 | 0.203 | -0.47 | -1.19, 0.24 | 0.196 |
| NAIP NDVI – 300 m buffer | 1.72 | -1.01, 4.45 | 0.218 | 1.59 | -1.13, 4.31 | 0.253 |
| NAIP NDVI – 500 m buffer | 2.12 | -0.74, 4.98 | 0.146 | 2.03 | -0.82, 4.88 | 0.162 |
