# Supplementary material for: Perceptions of green space usage, abundance, and quality of green space were associated with better mental health during the COVID-19 pandemic among residents of Denver
Source: PLoS One. 2022 Mar 2;17(3):e0263779. doi: 10.1371/journal.pone.0263779 (PMC8890647; doi:10.1371/journal.pone.0263779)
Supplement: S6 Table — (DOCX) [file pone.0263779.s007.docx]

|  | **CES-D-10 and sex** | | | **CES-D-10 and ethnicity** | | | **CES-D-10, sex, and ethnicity** | | |
| --- | --- | --- | --- | --- | --- | --- | --- | --- | --- |
| **Greenspace measure** | **Beta** | **95% CI** | **p-value** | **Beta** | **95% CI** | **p-value** | **Beta** | **95% CI** | **p-value** |
| “There is a lot of vegetation/greenery in my neighborhood” | | | | | | | | | |
| *Strongly Disagree* | — | — |  | — | — |  | — | — |  |
| *Disagree* | -1.53 | -2.91, -0.16 | **0.029** | -1.51 | -2.88, -0.13 | **0.032** | -1.50 | -2.88, -0.13 | **0.033** |
| *Agree* | -1.96 | -3.20, -0.71 | **0.002** | -1.99 | -3.23, -0.74 | **0.002** | -1.96 | -3.20, -0.72 | **0.002** |
| *Strongly Agree* | -2.59 | -3.93, -1.26 | **<0.001** | -2.59 | -3.92, -1.25 | **<0.001** | -2.58 | -3.91, -1.24 | **<0.001** |
| “I can see vegetation/greenery from my home” | | | | | | | | | |
| *Strongly Disagree* | — | — |  | — | — |  | — | — |  |
| *Disagree* | -0.42 | -1.92, 1.08 | 0.585 | -0.40 | -1.91, 1.10 | 0.598 | -0.42 | -1.93, 1.08 | 0.580 |
| *Agree* | -1.47 | -2.79, -0.15 | **0.029** | -1.50 | -2.82, -0.18 | **0.026** | -1.51 | -2.83, -0.19 | **0.025** |
| *Strongly Agree* | -1.88 | -3.26, -0.49 | **0.008** | -1.88 | -3.26, -0.49 | **0.008** | -1.89 | -3.28, -0.51 | **0.008** |
| “The nearest vegetated park/green space is easy for me to access” | | | | | | | | | |
| *Strongly Disagree* | — | — |  | — | — |  | — | — |  |
| *Disagree* | -2.32 | -4.72, 0.08 | 0.058 | -2.36 | -4.77, 0.04 | 0.054 | -2.36 | -4.76, 0.04 | 0.054 |
| *Agree* | -2.75 | -4.75, -0.74 | **0.007** | -2.81 | -4.82, -0.80 | **0.006** | -2.79 | -4.80, -0.78 | **0.007** |
| *Strongly Agree* | -3.04 | -5.05, -1.04 | **0.003** | -3.12 | -5.13, -1.11 | **0.002** | -3.09 | -5.10, -1.09 | **0.003** |
| “I spend a lot of time in spaces with natural vegetation” | | | | | | | | | |
| *Strongly Disagree* | — | — |  | — | — |  | — | — |  |
| *Disagree* | -2.68 | -4.19, -1.17 | **<0.001** | -2.62 | -4.13, -1.11 | **<0.001** | -2.74 | -4.25, -1.23 | **<0.001** |
| *Agree* | -3.47 | -4.92, -2.01 | **<0.001** | -3.43 | -4.88, -1.97 | **<0.001** | -3.56 | -5.02, -2.10 | **<0.001** |
| *Strongly Agree* | -3.90 | -5.43, -2.37 | **<0.001** | -3.79 | -5.32, -2.26 | **<0.001** | -3.97 | -5.50, -2.44 | **<0.001** |
| “The green spaces near my home are very high quality” | | | | | | | | | |
| *Strongly Disagree* | — | — |  | — | — |  | — | — |  |
| *Disagree* | -0.82 | -2.06, 0.42 | 0.196 | -0.84 | -2.09, 0.40 | 0.184 | -0.86 | -2.10, 0.39 | 0.176 |
| *Agree* | -1.30 | -2.48, -0.11 | **0.032** | -1.37 | -2.56, -0.18 | **0.024** | -1.33 | -2.52, -0.14 | **0.029** |
| *Strongly Agree* | -2.31 | -3.63, -0.99 | **<0.001** | -2.34 | -3.67, -1.02 | **<0.001** | -2.35 | -3.67, -1.02 | **<0.001** |
| NAIP NDVI – 300 m buffer | -4.54 | -9.54, 0.47 | 0.076 | -4.59 | -9.61, 0.43 | 0.074 | -4.76 | -9.78, 0.26 | 0.063 |
| NAIP NDVI – 500 m buffer | -5.52 | -10.76, -0.28 | **0.039** | -5.65 | -10.90, -0.39 | **0.036** | -5.77 | -11.02, -0.51 | **0.032** |
