# Supplementary material for: Perceptions of green space usage, abundance, and quality of green space were associated with better mental health during the COVID-19 pandemic among residents of Denver
Source: PLoS One. 2022 Mar 2;17(3):e0263779. doi: 10.1371/journal.pone.0263779 (PMC8890647; doi:10.1371/journal.pone.0263779)
Supplement: S7 Table — (DOCX) [file pone.0263779.s008.docx]

|  | **MMPI-2 and sex** | | | **MMPI-2, sex, and ethnicity** | | |
| --- | --- | --- | --- | --- | --- | --- |
| **Greenspace measure** | **Beta** | **95% CI** | **p-value** | **Beta** | **95% CI** | **p-value** |
| “There is a lot of vegetation/greenery in my neighborhood” | | | | | | |
| *Strongly Disagree* | — | — |  | — | — |  |
| *Disagree* | -1.86 | -4.71, 0.98 | 0.200 | -1.84 | -4.69, 1.01 | 0.206 |
| *Agree* | -2.67 | -5.24, -0.09 | **0.043** | -2.67 | -5.24, -0.09 | **0.043** |
| *Strongly Agree* | -5.09 | -7.87, -2.31 | **<0.001** | -5.08 | -7.86, -2.30 | **<0.001** |
| “I can see vegetation/greenery from my home” | | | | | | |
| *Strongly Disagree* | — | — |  | — | — |  |
| *Disagree* | -1.64 | -4.79, 1.52 | 0.309 | -1.65 | -4.80, 1.51 | 0.306 |
| *Agree* | -2.69 | -5.48, 0.09 | 0.058 | -2.73 | -5.52, 0.06 | 0.056 |
| *Strongly Agree* | -4.71 | -7.63, -1.78 | **0.002** | -4.72 | -7.65, -1.79 | **0.002** |
| “The nearest vegetated park/green space is easy for me to access” | | | | | | |
| *Strongly Disagree* | — | — |  | — | — |  |
| *Disagree* | -4.42 | -9.33, 0.48 | 0.077 | -4.47 | -9.38, 0.43 | 0.074 |
| *Agree* | -4.95 | -9.09, -0.82 | **0.019** | -5.01 | -9.14, -0.87 | **0.018** |
| *Strongly Agree* | -7.13 | -11.26, -3.00 | **<0.001** | -7.19 | -11.32, -3.06 | **<0.001** |
| “I spend a lot of time in spaces with natural vegetation” | | | | | | |
| *Strongly Disagree* | — | — |  | — | — |  |
| *Disagree* | -6.06 | -9.16, -2.96 | **<0.001** | -6.16 | -9.27, -3.06 | **<0.001** |
| *Agree* | -8.05 | -11.04, -5.06 | **<0.001** | -8.20 | -11.21, -5.19 | **<0.001** |
| *Strongly Agree* | -9.39 | -12.54, -6.23 | **<0.001** | -9.51 | -12.67, -6.35 | **<0.001** |
| “The green spaces near my home are very high quality” | | | | | | |
| *Strongly Disagree* | — | — |  | — | — |  |
| *Disagree* | -2.24 | -4.82, 0.33 | 0.088 | -2.27 | -4.85, 0.30 | 0.084 |
| *Agree* | -3.67 | -6.14, -1.20 | **0.004** | -3.69 | -6.16, -1.22 | **0.004** |
| *Strongly Agree* | -6.06 | -8.80, -3.32 | **<0.001** | -6.09 | -8.83, -3.34 | **<0.001** |
| NAIP NDVI – 300 m buffer | -7.17 | -17.69, 3.35 | 0.182 | -7.40 | -17.95, 3.15 | 0.170 |
| NAIP NDVI – 500 m buffer | -8.04 | -19.04, 2.97 | 0.153 | -8.30 | -19.34, 2.74 | 0.141 |
