# Supplementary material for: Perceptions of green space usage, abundance, and quality of green space were associated with better mental health during the COVID-19 pandemic among residents of Denver
Source: PLoS One. 2022 Mar 2;17(3):e0263779. doi: 10.1371/journal.pone.0263779 (PMC8890647; doi:10.1371/journal.pone.0263779)
Supplement: Attachment — Submitted filename: Response to Reviewers.docx [file pone.0263779.s009.docx]

**Response to Reviewers**
*We have revised our manuscript to respond to the edits of the reviewers and editor and we thank them for their thoughtful comments that we believe have improved out paper. Our responses to each comment are below in italics below each comment. Additionally, we document where in the manuscript the reviewer can find any associated changes in the text. The line numbers refer to the line numbers of the word document with tracked changes.*

*Additionally, we have placed all of the data in a repository by our university and will soon be receiving the doi for that data that we can provide to Plos One to include with our manuscript.*

Reviewer #1: This paper uses survey results to further our understanding of the potential for green space to provide urban residents with mental health benefits. Especially in the context of the COVID-19 pandemic this is an important topic that the authors have much to contribute to. The inclusion of multiple measures of green space, multiple mental health measures, and pandemic related stressors presents and improvement on previous research that fails to account for this wide range of influences and impacts.
*Thank you for this comment.*

However, there remain several major issues that need to be addressed before the manuscript is suitable for publication, specifically:
• The survey results are compared between the different time periods, but with different respondents in each period, it might simply be a result of who is responding rather than any wider differences among Denver residents

*We agree that this is possible and have added in the following “*These differences could be true differences or they could be due to different people responding to our survey during different time periods.*” on lines 405-406 in the edited manuscript.*

• There is no comparison of the respondents (which seem to skew whiter and wealthier) with Denver’s population. Which do the results speak to?

*Thank you for pointing this out. We have added in a supplemental table (S2 Table) that shows our population percentages by demographic characteristics compared to Denver as a whole and the neighborhoods that were targeted in our initial screening. We additionally state in the results section on lines 352-356:*

*“*Respondents to our survey were more likely to be female, older, White, and not Hispanic/Latino than the population of Denver or the neighborhoods in Denver that we targeted. They were also more likely to have a Bachelor’s degree, and a higher income than the average in Denver or the neighborhoods we targeted, but they were about equally likely to have health insurance as the population of Denver (S2 Table). *”*

*We also added the following related sentence to our discussion section on lines 641-643: “*Our study population differs from the city of Denver in key demographic variables which we adjusted for in our analyses but does limit the generalizability of the findings.”

o Similarly, I didn’t see a response rate anywhere in the MS (apologies if I missed it)

*Thank you for pointing out this omission. We have added in our response rate and completion rate into the section on the study population on lines 340-341.*

• The policy implications need to be more developed-what can policymakers and managers take away from this research?

*Thank you. We have put in a few more sentences at the end of the discussion about policy implications of this and other work on the mental health benefits of green spaces particularly during the pandemic.*

Other less pressing issues include:
• The single sentence paragraph at the end of the discussion section (Lines 591-593) should be integrated with previous paragraph or cut

*Thank you – we have added more to this paragraph on policy implications which deals with this issue as well as the previous comment. This section is now on lines 655-665 and reads:*

*“*Our research provides evidence to support municipal policies that do more than just add more green space (abundance), but that also work to improve the perceived quality and usage of green space by residents. Working with community groups to determine what denotes better quality of green space and what makes people more likely to use green space is an important area of future research. Given the growing evidence of mental health benefits of green space [13] and the growing evidence of benefits of green space for mental health during the pandemic highlighted by this and other studies [14,20,23–25], it is essential that policies are enacted to ensure that green spaces are available to residents during the rest of this pandemic and during future pandemics or other societal challenges. Additionally, our research adds to a growing body of evidence, which includes evidence of plausible biological mechanisms and from experimental studies [64], documenting mental health benefits of green space generally.”

• It would be useful for the reader unfamiliar with Denver’s park system to get a description of the city’s greenspace and who has access to it

*We have added a section on the park system and what is known about it in the “Study Population” section found on lines 143-145. This sentence says: “*Denver is an interesting city from which to study green space exposure and health because of previous studies documenting unequal access to parks by race/ethnicity in Denver [42] and lower levels of vegetation, as measured by satellites, in lower income areas of Denver [43].”

• A justification of the distance buffers used to measure “objective” green space exposure would be useful-even just citations of previous studies that use similar distances

*We have added to lines 292-296 this statement, “*There is no agreement on what radial buffer size is most appropriate in green space and health studies. Different studies have found that both larger and smaller buffers are better, but despite this disagreement, the clearest evidence is that the buffer size matters [55,56]. We chose these buffer sizes because they are commonly used in green space and mental health research [57].”

• The survey delivery method is introduced (Lines 221-222) before the survey is finished being described, I would move it down after the perceived green space paragraph/section

*Thank you, we have done this.*

Reviewer #2: Fundamentally it is a good paper. It is an important question and the authors have nicely marshalled a unique set of data to offer insights.

I have one main concern, and that is about self-selection. Humans with certain attributes would tend to self-select themselves to live in places with greater access to vegetation. These attributes could easily be correlated with some of the phenomena of interest. Thus, this estimation strategy runs the risk of endogeneity bias. As an example, there's a well known correlation between physics researchers and the mountains.

*Thank you for this comment. We agree that this is a potential concern that individuals with better mental health may choose to live in greener areas, which could indicate a self-selection bias that would lead to reverse causality whereby mental health is causing green space exposure rather than green space exposure causing better mental health. This self-selection bias, to our knowledge, has not been assessed for green space exposure and mental health outcomes, although at least one study disproved it for green space exposure and physical activity or obesity measures* (4)*.*

*One of the ways that we adjusted for the potential that people with certain characteristics may be more likely to live in places with more vegetation was to adjust for factors that we collected that could be such factors such as income and educational attainment. We have also now addressed this concern in our discussion section on lines 641-645 as follows:*

*“*There is also the potential that our findings suffer from self-selection bias, whereby people with better mental health are more likely to choose to live in greener areas. Because our study is cross-sectional, we could not look at how people choose to move related to these characteristics, but we did adjust for factors that are known to be associated with differential exposure to green space such as income, educational attainment, and race/ethnicity.”

The research strategy adopted by the authors will inevitably face these constraints. The writing of the paper should be more careful to stop short of making causal claims, and raise the concern that these correlations could be in fray.

*Thank you for this. We have reread and edited our paper to ensure that we are not making causal statements. We already had this statement in our discussion: “*Although our study has responses from various times in the pandemic, we only have one response per person, thus our study is cross-sectional which limits our ability to consider the results causal” (on lines 629-631 now), *but have now made editorial changes – most notably to our title – to ensure that we are not overstating our findings.*

 When submitting your revision, we need you to address these additional requirements.

1. Please ensure that your manuscript meets PLOS ONE's style requirements, including those for file naming. The PLOS ONE style templates can be found at 
<https://journals.plos.org/plosone/s/file?id=wjVg/PLOSOne_formatting_sample_main_body.pdf> and 
<https://journals.plos.org/plosone/s/file?id=ba62/PLOSOne_formatting_sample_title_authors_affiliations.pdf>

*We have reviewed our formatting and believe that we have made the necessary changes to align our manuscript with the style templates provided.*

2. Thank you for stating the following in the Acknowledgments Section of your manuscript:

"The CUPC is supported by funds from the Eunice Kennedy Shriver National Institute of Child Health & Human Development of the National Institutes of Health (NIH) under Award Number P2CHD066613. The content is solely the responsibility of the authors and does not necessarily represent the official views of the NIH"

We note that you have provided funding information that is not currently declared in your Funding Statement. However, funding information should not appear in the Acknowledgments section or other areas of your manuscript. We will only publish funding information present in the Funding Statement section of the online submission form. 
Please remove any funding-related text from the manuscript and let us know how you would like to update your Funding Statement. Currently, your Funding Statement reads as follows: 
 
"Research reported in this publication was supported by the Harvard JPB Environmental Health Fellowship (CER) and the Developmental Core of the University of Colorado Population Center (CUPC) (CER). The CUPC is supported by funds from the Eunice Kennedy Shriver National Institute of Child Health & Human Development of the National Institutes of Health (NIH) under Award Number P2CHD066613. The content is solely the responsibility of the authors and does not necessarily represent the official views of the NIH. NIH and CUPC did not play a role in the study design, data collection and analysis, decision to publish, or preparation of the manuscript. Researchers at Harvard provided feedback on the study design, but the JPB funders did not. Neither JPB nor Harvard played a role in the data collection and analysis, decision to publish, or preparation of the manuscript."

*We have removed the funding statement from the text and have updated the funding statement in the resubmission.*

**References**

1.	Reid CE, Kubzansky LD, Li J, Shmool JL, Clougherty JE. It’s not easy assessing greenness: A comparison of NDVI datasets and neighborhood types and their associations with self-rated health in New York City. Health Place. 2018 Sep 21;54:92–101.

2.	James P, Berrigan D, Hart JE, Hipp JA, Hoehner CM, Kerr J, et al. Effects of buffer size and shape on associations between the built environment and energy balance. Health & place. 2014 May;27:162–70.

3.	Davis Z, Guhn M, Jarvis I, Jerrett M, Nesbitt L, Oberlander T, et al. The association between natural environments and childhood mental health and development: A systematic review and assessment of different exposure measurements. Int J Hyg Environ Health. 2021 May 11;235:113767.

4.	James P, Hart JE, Arcaya MC, Feskanich D, Laden F, Subramanian SV. Neighborhood Self-Selection: The Role of Pre-Move Health Factors on the Built and Socioeconomic Environment. International Journal of Environmental Research and Public Health. 2015 Oct;12(10):12489–504.
